# What Role do Hurricanes Play in Sediment Delivery to Subsiding River Deltas?

**DOI:** 10.1038/srep17582

**Published:** 2015-12-02

**Authors:** James E. Smith, Samuel J. Bentley, Gregg A. Snedden, Crawford White

**Affiliations:** 1 Department of Geology and Geophysics and Coastal Studies Institute, Louisiana State University, Baton Rouge, LA 70803 USA; 2 U.S. Geological Survey, National Wetlands Research Center, Baton Rouge LA 70803 USA

## Abstract

The Mississippi River Delta (MRD) has undergone tremendous land loss over the past century due to natural and anthropogenic influences, a fate shared by many river deltas globally. A globally unprecedented effort to restore and sustain the remaining subaerial portions of the delta is now underway, an endeavor that is expected to cost $50–100B over the next 50 yr. Success of this effort requires a thorough understanding of natural and anthropogenic controls on sediment supply and delta geomorphology. In the MRD, hurricanes have been paradoxically identified as both substantial agents of widespread land loss, and vertical marsh sediment accretion. We present the first multi-decadal chronostratigraphic assessment of sediment supply for a major coastal basin of the MRD that assesses both fluvial and hurricane-induced contributions to sediment accumulation in deltaic wetlands. Our findings indicate that over multidecadal timescales, hurricane-induced sediment delivery may be an important contributor for deltaic wetland vertical accretion, but the contribution from hurricanes to long-term sediment accumulation is substantially less than sediment delivery supplied by existing and planned river-sediment diversions at present-day river-sediment loads.

The Mississippi River Delta (MRD) has undergone tremendous land loss over the past century due to natural and anthropogenic influences^1^, a fate shared by many river deltas globally^2^. The MRD is now the subject of a massive and globally unprecedented program to sustain remaining subaerial portions of the delta^3^ that will cost $50–100 billion over >50 yr^3^. In order for this program to succeed, we must fully understand both natural and anthropogenic controls on sediment supply and delta geomorphology^2^,^4^,^5^. In the MRD, hurricanes have been paradoxically identified as substantial agents of both widespread land loss^6^, and vertical marsh sediment accretion^7^,^8^,^9^. We present the first multi-decadal chronostratigraphic assessment of sediment supply for a major coastal basin of the MRD that assesses both fluvial and hurricane-supplied sediment contributions to vertical delta accretion. Our sediment calculations indicate that over multidecadal timescales, hurricane-induced sediment delivery may be an important component for deltaic wetland vertical accretion, but the long-term sediment accumulation rate from hurricanes is substantially less than sediment delivery supplied by existing and planned river-sediment diversions at present-day river-sediment loads.

The subaerial and coastal regions of river deltas are among the world’s most intensely managed and modified landscapes^10^. Most existing deltas developed over the past 5,000–7,000 years, after global sea levels became relatively static compared with their rapid rise in the early Holocene, allowing fluvial sediments to prograde seaward from stable shorelines^10^, forming broad, low-gradient coastal plains^5^. With over 500 million people currently residing in deltas worldwide, rising sea levels are beginning to place human habitation, infrastructure, and economies at risk^2^,^10^, making sustainability of deltaic landscapes a global problem.

The elevation and area of coastal deltaic wetlands tend to evolve towards equilibrium with the supply of river sediment, production of organic soils, local subsidence, and eustatic sea-level rise^4^, so conservation strategies for deltaic wetlands must take these processes into account. In the MRD, like many other deltas, sediment supply has been reduced by human activities^1^,^5^, subsidence rates can exceed 25 mm yr^−1^^11^, and sea-level rise is accelerating. These combined trends have contributed to the loss of nearly 4000 km^2^ of MRD coastal wetlands since 1932, with over 50% of that loss being directly attributed to submergence^12^.

The long-term sustainability of the MRD is of great national and international importance due to the high value of associated communities, natural resources, and commerce^13^. In the MRD, river-sediment diversions are being implemented as key components of a comprehensive restoration plan^3^ to reconnect the sediment supply that once created and maintained MRD wetlands. The impacts of existing MRD diversions over the past 20 years have been disputed, with some studies showing increased organic and mineral wetland accretion near diversions^14^,^15^,^16^. Diversions have also been implicated in land loss, especially following Hurricane Katrina in 2005, during which over 100 km^2^ of wetlands were converted to open water in upper Breton Sound^6^, into which the Caernarvon Freshwater Diversion (CFD) has discharged Mississippi River water for over 20 years. This land loss has been linked to diversion inflows of nutrient-rich river water, associated with weakened soils and plant growth^17^,^18^,^19^.

Paradoxically, hurricanes have also been identified as sources of sediment for vertical wetland accretion, via surge inundation, with estimates of 116–131 million metric tons (Mmt) of Louisiana coastal wetland sedimentation from hurricanes Katrina and Rita in 2005, prompting the suggestion that hurricanes are more constructive than river-sediment supply to MRD wetland growth^7^,^8^,^9^. The applicability of these findings to coastal restoration has been debated^20^,^21^, but tropical cyclones are clearly important drivers of coastal geomorphology, and may play an important role in wetland sedimentation and the ability of marshes to keep pace with sea-level rise.

To clarify the relative roles of fluvial versus hurricane-supplied sediment to vertical wetland accretion, this study integrates hurricane landfall records since 1946 with ^137^Cs geochronology, mineral content and bulk density obtained from 27 cores collected across wetlands in Breton Sound, Louisiana (Fig. 1). Hurricane event layers were identified as widely distributed layers of elevated mineral sediment content, with ages (from ^137^Cs geochronology) comparable to known hurricane landfalls. We document spatial and temporal patterns of hurricane-induced sediment accumulation across the basin, and place these rates in context with rates of direct fluvial sediment input to the region over the same time span.

## Study Area

**Figure 1:**
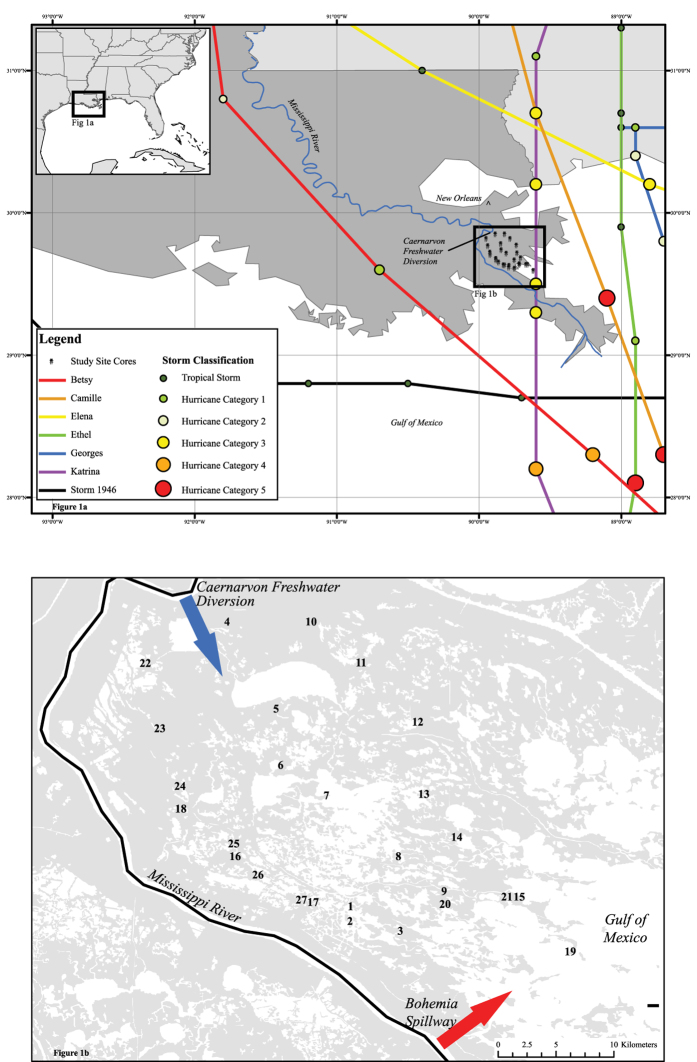
(**a**) Map of the study area, showing the locations of the 27 coring sites and the trajectories of the seven category 3+ storms known to have made landfall within 100 km of Breton Sound basin. (**b**) Locations of the 27 coring sites and also the Caernarvon Freshwater Diversion (CFD) and the Bohemia Spillway, two sources of significant fluvial inputs. This figure was prepared by the authors, using ESRI ArcGIS^©^ software, ESRI geodata that are royalty-free for public use, and the NOAA HURDAT database (http://www.aoml.noaa.gov/hrd/hurdat) that is a US government data source and royalty-free for public use.

The Breton Sound estuary system encompasses roughly 1000 km^2^ of fresh, brackish, and saline marshes located southeast of New Orleans and east of the Mississippi River (Fig. 1a). The region is underlain by Holocene Mississippi Delta deposits of the St Bernard and Plaquemines lobes of the Mississippi Delta, that were active 4,000–2,000 years ago and 1,000–500 years ago, respectively^22^. Direct Mississippi River water and sediment sources to Breton Sound include the CFD and the Bohemia Spillway (Fig. 1b). The CFD was designed to deliver freshwater at a maximum rate of 225 m^3^ s^−1^, while the average flow since operations started in 1991 has been 40 m^3^ s^−1^^23^. Located on the southwestern boundary of the basin, the Bohemia Spillway also contributes significant fluvial sediments to Breton Sound, with average water discharge rates of 32 m^3^ s^−1^ and an annual sediment load for 2008–2010 of 0.3 Mmt yr^−1^^24^, but longer term loading rates are not documented.

## Results

Vertical accretion rates determined from analysis of ^137^Cs activity are 0.24–1.0 cm yr^−1^ (Supplementary Table S1). Time windows for 74 of the 101 layers of elevated mineral content identified in core (*m*_*z*_, equations [1,2,3] in Methods) overlapped with the passage of seven category 3+ hurricanes within 100 km of the Breton Sound basin over the past 70 years (Unnamed, 1946; Ethel, 1960; Betsy, 1965; Camille, 1969; Elena, 1985; Georges, 1998; Katrina, 2005)^25^.

**Figure 2:**
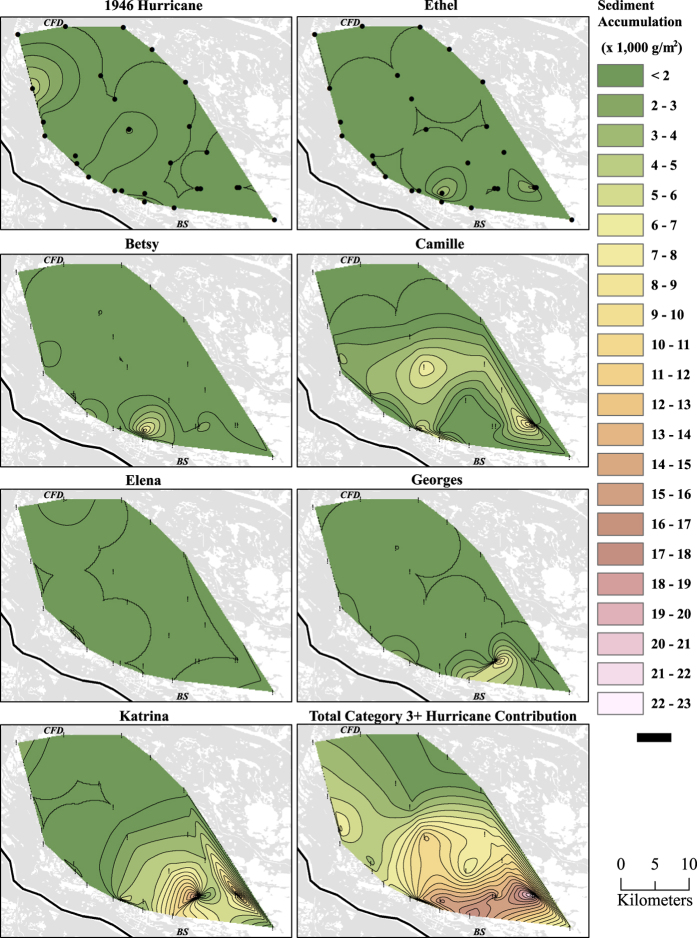
Lateral interpolation of hurricane-induced sediment accumulation across the 518 km^2^ region encompassed by the 27 cores for each of the category 3+ hurricanes and the accumulation attributable to the sum of all seven hurricanes. Blue arrows labeled “CFD” mark location of the Caernarvon Freshwater Diversion, and red arrows labeled “BS” are just north of the Bohemia Spillway, which falls just outside the figure boundaries. Data used for making this figure are presented in Supplementary Table S1.

Rates of mineral sediment accretion (*MSA,* equation [6] in Methods) from the seven hurricanes recorded in 27 cores were highly variable, ranging from 40 g  m^−2^ (Elena) to 16,300 g  m^−2^ (Katrina; Supplementary Table S1). Discernable spatial patterns of hurricane-induced *MSA* were evident for Camille, with a central region of elevated *MSA*; and Georges and Katrina, with elevated MSA near the marine end of the basin, gradually decreasing towards inland coring sites (Fig. 2). Total hurricane-related *MSA* also showed a gradual reduction with increasing distance inland from the seaward extent of the subaerial marshes of the remnant delta (Fig. 2).

Individual core data for each event were laterally interpolated and summed to estimate total hurricane-induced sediment accumulation (and uncertainty) for each hurricane across the entire 518 km^2^ region encompassed by the 27 cores. Results ranged from 0.1 ± 0.08 Mmt (Elena) to 1.19 ± 0.47 Mmt (Katrina), with a total of 3.38 ± 1.68 Mmt attributable to the sum of all seven category 3+ storms combined (Table 1). Sedimentation in this region associated with undesignated events was estimated to be 0.89 ± 0.41 Mmt, and total mineral sediment content in the region was 31.03 Mmt. Subtracting the total storm-induced and undesignated event sedimentation from the total mineral sediment content leaves a ‘residual’ sediment content of 26.76 Mmt in the 518 km^2^ region (Table 1).

## Discussion

### Sources of Fluvial Sediment to Breton Sound

Mean rates of vertical accretion throughout Breton Sound obtained through analysis of ^137^Cs in this study (0.77 cm yr^−1^) are corroborated by previous basin-wide studies of vertical accretion in Breton Sound (0.72 cm yr^−1^)^14^ and other regions of the Mississippi River delta plain (0.78 cm yr^−1^)^16^. Taking the total mineral sediment accumulation in the 518 km^2^ study area (31 Mmt) and dividing it by the average timespan of all sediment cores (64 y) yields an annual regional mineral accumulation rate of 0.48 Mmt y^−1^ (Table 1). This rate is relatively modest compared to the West Bay diversion, located downstream on the Mississippi River, which receives 6% of total Mississippi flow, and accumulates up to 2 Mmt of sediment during <1 yr within a 70 km^2^ receiving basin^26^.

Direct fluvial input into the upper Breton Sound basin did not occur until the CFD commenced operations in 1991 (except for three months in 1927 during a large Mississippi River flood event). Assuming a constant annual sediment discharge rate of 0.1 Mmt y^−1^^23^ through the CFD since 1991, it may have delivered the equivalent of 21% of the total sediment accumulation in the 518 km^2^ study area over the past 23 years. The proportion of sediment delivered via the CFD that is sequestered within the basin remains unclear. Long-term sediment discharge from the Bohemia Spillway is not known, but as this is a relatively natural feature, the 0.3 Mmt yr^−1^ sediment discharge observed for 2008–2010^24^ may be applicable to our 64-year study time span. If so, then annual sediment delivery to the Breton Sound basin through the Bohemia Spillway is equivalent to 62% of total annual mineral sediment accumulation in the 518 km^2^ study area encompassed by the 27 coring sites within Breton Sound.

Downstream of the Bohemia Spillway, within 40 km of the marshes in our study area, 27% of the Mississippi River’s total annual discharge escapes through eight additional river outlets each year contributing 19.9 Mmt yr^−1^ of mineral sediments into the shallow marine bays of Breton Sound^24^. These sediments are discharged downbasin of the Breton Sound wetlands, but still provide a large potential sediment reservoir that could be delivered inland by a variety of processes capable of inundating the basin’s wetlands, including tides, frontal passages, and tropical cyclones^27^. Routine flooding associated with lunar tides and cold-front winds may drive the sediment accumulation captured in our residual term (Table 1).

### Comparison with Short-Term Hurricane-generated Sediment Deposition

The spatially averaged event sedimentation rate in Breton Sound for hurricane Katrina (sampled weeks after landfall) was previously estimated to be 26,000 ± 13,000 g cm^−2 34^ (see Methods), comparable to regionally averaged event sedimentation rates (including areas beyond Breton Sound) estimated for hurricanes Rita (2005) and Gustav (2008)^8^,^9^. Accumulation rates per unit area for Katrina in this study were an order of magnitude less. Unlike previous estimates, our samples were collected three to eight years after landfall. It is important to note that our estimates and those cited above represent “net” vertical accretion rates, measuring sediment accumulation that contributes to wetland elevation only. These results do not account for sediment bypass, loss of sediment through lateral or surficial erosion and export to shelf margins during hurricane (or other) sediment transport events.

**Figure 3:**
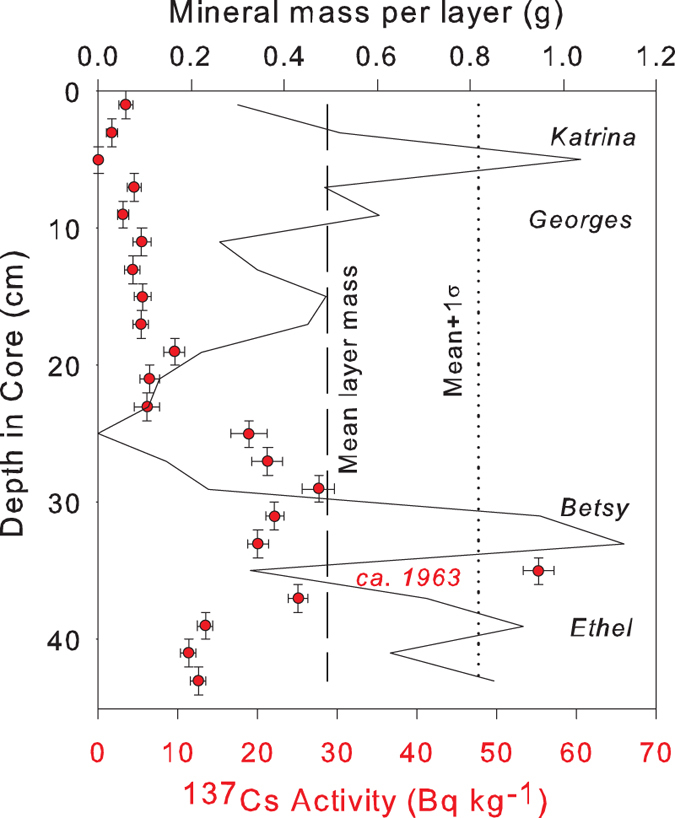
Vertical profiles of ^137^Cs activity (points) and mineral sediment mass (solid line) for core obtained at site 16. Mean mineral mass (dashed vertical line) and mean mineral mass plus one standard deviation (dotted vertical line) are also shown.

A possible explanation for this order of magnitude difference between our results and the earlier Breton study^7^ is that up to 90% of sediment mass delivered by hurricane Katrina may have been vertically reworked to the extent that it is indistinguishable from routine mineral sediment contributions associated with tidal and winter storm inundation. However, because mineral mass peaks in our core data are clearly defined (Fig. 3), present across many sites throughout the basin, and coincident with passage of several category 3+ hurricanes dating back to 1946 (Fig. 3), it does not seem reasonable that mineral mass peaks in our data would have attenuated by 90% in such a short time span. It is possible that our methods have underestimated mass accumulation per storm to some degree, but equally likely that other processes unrelated to hurricanes are critically important for providing sediment input to the marshes in Breton Sound basin.

Another possible explanation for differences between our decade-averaged results and the event-scale measurements in previous studies relates to methodology. Previous results for Breton Sound hurricane Katrina sedimentation are all non-zero values^7^, whereas our spatially weighted results for the Katrina event layer include sizeable areas of low or zero sediment accumulation (Fig. 2). These factors make quantitative comparison of our Katrina results with previous estimates challenging. More generally, rather than portray recent mass accumulation and the extent of hurricane event layers across Louisiana coast immediately after a single landfall event, our data readily show hurricane contributions post-deposition and their role in the long-term vertical accretion of the marsh over seven decades, and document often-observed differences between short-term deposition and long-term sediment accumulation rates^28^.

While our results estimate mineral sedimentation in the Breton Sound basin that coincided with seven major hurricane landfall events between 1946 and 2005, they do not provide information regarding the source of these sediments. Previous studies of hurricane-induced sedimentation have indicated bed sediments in lakes and bays at the seaward end of the coastal basins may serve as sediment sinks over decadal timescales which can become sources available for upbasin transport onto coastal marsh surfaces during extreme conditions typically accompanying storm surges^29^. Other studies suggest a combination of nearshore and fluvial sediment sources for hurricane-induced sedimentation^30^. In our study, it is plausible that sediments observed in the core event layers were derived from multiple sources, including (1) nearshore bay bottom bed sediments that are heavily subsidized by fluvial materials discharged through the eight river outlets below the Bohemia Spillway, (2) sediments transported upbasin from the mouth of the Mississippi River, (3) marsh sediments eroded from one location and deposited in another, and (4) sediments introduced into the head of the estuary via the CFD. Further investigation using paleoindicators and geochemical markers is needed to determine the relative importance of these sources.

In conclusion, our results demonstrate that local fluvial sediment supply, redistributed via regular flooding from river floods, tides, frontal passages, and other events, is sufficient to account for mineral mass in vertically accreting wetlands, despite the findings of some recent studies. Further, the long-term history of mineral sediment accretion in these wetlands clearly records the impacts of hurricane sediment delivery, but is not dominated by hurricanes, such that other sediment delivery processes must be equally influential on deltaic wetland morphodynamics.

## Methods

### Sample Collection and Mineral Peak Analysis

A total of 27 sediment cores were collected and analyzed for this study; 18 were collected during Jan-Feb 2008 and the remaining nine in Feb 2013. Cores were collected with 10-cm diameter, thin-walled aluminum pipes 60 cm in length. Compaction was measured before extraction, and was <15% for all cores. Cores were stored frozen prior to processing, during which partially thawed cores were extruded whole from the aluminum pipe, measured, and sliced into 2-cm sections (∆*z* = 2 cm) using an industrial band saw. Sections were further divided radially into two subsamples of known volume, where 75% of the material was reserved for radiochemistry, and 25% for loss-on-ignition and dry bulk density analysis. Because the methods below are based on mass accumulation at known points in time, rather than linear sediment accumulation rates, results are relatively insensitive to the possible effects of sediment compaction during core collection or dilation during freezing.

Mineral (*Φ*_*S*_) fractions were determined using standard loss-on-ignition methods^31^. Dry bulk density (*ρ*_*b*_, g cm^−3^) was derived from the mass and total volume of the sample after drying the sample at 105 °C for 24 hours. Mineral mass per unit area per core section (*m*_*z*_, g cm^−2^) was then determined as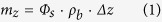 where *Δz* is 2 cm, or the core section thickness. Core profiles of *m*_*z*_ were used to calculate whole-core core averages (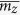) and standard deviations (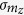) of mineral mass (Fig. 3).

### Geochronology

The larger subsample of each interval was reserved for determination of vertical accretion rates (VARs) using gamma spectrometry analysis of ^137^Cs^14^. Subsamples were dried at 105 °C for 24 hours, water content was assessed, and subsamples were then ground, placed into plastic petri dishes, then sealed and measured for ^137^Cs activity at 661.3 KeV on a shielded high-purity germanium detector for 24 hours. An anthropogenic radioisotope, ^137^Cs was first introduced to the atmosphere in 1954 by nuclear bomb testing. Atmospheric ^137^Cs levels peaked in 1963–1964 and dropped to insignificant levels by 1980; thus, the core section with the greatest ^137^Cs activity should correspond to soil exposed to the atmosphere during 1963. Assuming negligible bioturbation, VARs (cm yr^−1^) for each core were calculated as the depth of the peak in ^137^Cs activity (*Z*_*max*_) divided by the time since 1963.

### Combining Geochronology and Mineral Mass Variations to Determine Storm-Sediment Contributions

Peaks in mineral content, defined as depth ranges where *m*_*z*_ >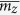, were used to identify event strata. Year of deposition *year*_*peak*_ and uncertainty of *σ*_*peak*_ for these mineral content peaks were estimated from the depth of maximum mineral content per event layer (*z*_*hur*_) as 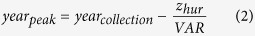

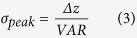


For each peak in mineral content in each core, a time window defined as *year*_*peak*_ ± *σ*_*peak*_ was compared to known chronology of post-1946 category 3+ hurricanes passing within 100 km of the Breton Sound basin^25^. Peaks in mineral mass were then assigned to known category 3+ hurricanes if their estimated age window encompassed the landfall year of a particular storm. Mineral mass peaks which did not fall within the age of a known category 3+ hurricane were categorized as “undesignated events”, indicating that although they appeared to be linked to distinct depositional events, the peaks could not be temporally linked via ^137^Cs dating techniques to any specific category 3+ storm that made landfall within 100 km of the study area. Total mineral sediment content for each core was calculated by summing *m*_*z*_ values for all sections of the core. Residual sediment content for each core, defined as the total mineral content minus that attributable to storm and undesignated events, was also calculated.

### Storm Mass Contributions

Mineral mass peaks were rarely restricted to one vertical core section; more often they encompassed multiple sections. For each recognized storm event layer in a given core, we estimated the upper bound of mineral sediment accumulation *MSA*_*up*_ attributable to the layer’s designated storm as the difference between *m*_*z*_ and 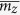 for each section in the storm layer *i*, summed over all sections in the storm event layer, or
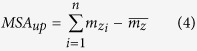
 where *n* is the total number of core sections in the storm event layer. Similarly, the lower bound of mineral sediment accumulation *MSA*_*lo*_ was estimated as
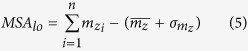
 where *σm*_*z*_ is the whole-core standard deviation of *m*_*z*_. Equations (4) and (5) serve to bracket reasonable upper and lower bounds of the true mineral mass contribution per event layer within a single core *MSA*, taken as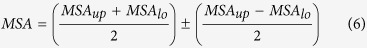 where the second term on the right-hand side is the uncertainty of the estimate (Table 1).

Natural neighbor interpolation using GIS software^32^ was conducted on the *MSA* values (equation [6]) and *MSA* range per layer (equations [4 and 5]) obtained from each storm at the 27 coring sites to estimate spatially continuous mineral mass contribution from each storm event layer across a 518 km^2^ polygon in Breton Sound basin (roughly 50% of the total basin area) and to reveal spatial patterns of storm-induced sedimentation. Interpolated values were obtained for each pixel, converted from g cm^−2^ to Mmt km^−2^ (million metric tons per km^2^), and pixel values were then summed across the study to provide estimates of total sediment input attributable to each storm event for the polygon.

For comparison of our *MSA* data with previous event sedimentation from Hurricane Katrina^7^, individual event-sedimentation data from Breton Sound samples^7^ were averaged first by station, then with spatial weighting to produce a Breton Sound average and standard deviation.

## Additional Information

**How to cite this article**: Smith, J. E. *et al*. What Role do Hurricanes Play in Sediment Delivery to Subsiding River Deltas? *Sci. Rep.* **5**, 17582; doi: 10.1038/srep17582 (2015).

## Supplementary Material

Supplementary Information

## Figures and Tables

**Table 1:** Laterally interpolated sediment accumulation rates for category 3+ hurricane events and undesignated depositional events identified in this study.

**Sediment Source**	**Sediment Accumulation**		**Annual Sediment Accumulation Rate**		**% Sediment Inventory**
	**g m**^**−2**^	**Mmt**	**g m**^**−2**^**y**^**−1**^	**Mmt y**^**−1**^	
Katrina	2300 ± 900	1.19 ± 0.47	–	–	3.8
Georges	640 ± 380	0.33 ± 0.20	–	–	1.1
Elena	190 ± 160	0.10 ± 0.08	–	–	0.3
Camille	1980 ± 840	1.03 ± 0.44	–	–	3.3
Betsy	630 ± 390	0.33 ± 0.20	–	–	1.1
Ethel	250 ± 390	0.13 ± 0.09	–	–	0.4
1946	540 ± 170	0.28 ± 0.21	–	–	0.9
Total Cat 3+ Storm	6530 ± 3240	3.38 ± 1.68	102	0.05	10.9
Undesignated Events	1720 ± 790	0.89 ± 0.41	27	0.01	2.9
Residual	51653	26.76	804	0.42	86.2
Total Sediment Inventory	59903	31.03	933	0.48	100.0

The residual represents the difference between total mineral sediment inventory and the event-driven accumulation. The intervals about the values indicate the upper and lower bounds of the accumulation estimates through lateral interpolation of *MSA*_*up*_ and *MSA*_*lo*_ (see equations 4, 5, 6).
