# Supplementary material for: What Role do Hurricanes Play in Sediment Delivery to Subsiding River Deltas?
Source: Sci Rep. 2015 Dec 2;5:17582. doi: 10.1038/srep17582 (PMC4667243; doi:10.1038/srep17582)
Supplement: Supplementary Information [file srep17582-s1.doc]

**What Role do Hurricanes Play in Sediment Delivery to Subsiding River Deltas?**

Supplementary Data Set

James E. Smith IV^1^, Samuel J. Bentley, Sr.^1^, Gregg A. Snedden^2^, Crawford White^1^

1. Department of Geology and Geophysics and Coastal Studies Institute, Louisiana State University, Baton Rouge, LA 70803 USA

2. U.S. Geological Survey, National Wetlands Research Center, Baton Rouge LA 70803 USA

* corresponding author sjb@lsu.edu

Supplementary Table S1. Core locations, and event-layer *MSA*, total hurricane *MSA* (used to create Fig. 2) and undesignated event *MSA* per core, and residual *MSA*.

| Core Id | Lat. N (deg.) | Long. W(deg.) | SAR (cm/yr) | Uncertainty of time window (y) | Katrina 2005 (g/m^2^) | Georges 1998 (g/m^2^) | Elena 1985 (g/m^2^) | Camille 1969 (g/m^2^) | Betsy 1965 (g/m^2^) | Ethel 1960 (g/m^2^) | Unnamed 1946 (g/m^2^) | Undesig. event total (g/m^2^) | Total local hurricane MSA  (g/m^2^) | total MSA (g/m^2^) | Residual MSA (g/m^2^) |
| --- | --- | --- | --- | --- | --- | --- | --- | --- | --- | --- | --- | --- | --- | --- | --- |
| 1 | 29.66392 | -89.8741 | 0.58 | 3.4 | 2,055 | 0 | 3,125 | 0 | 0 | 0 | 0 | 2,115 | 5,180 | 45,814 | 40,634 |
| 2 | 29.64639 | -89.8588 | 0.73 | 2.7 | 0 | 1,523 | 0 | 0 | 2,841 | 0 | 1,837 | 0 | 6,201 | 48,352 | 42,151 |
| 3 | 29.70044 | -89.9164 | 0.70 | 2.9 | 338 | 2,095 | 38 | 1,033 | 0 | 59 | 423 | 0 | 3,986 | 24,132 | 20,146 |
| 4 | 29.8449 | -89.8894 | 0.56 | 3.6 | 0 | 0 | 2,072 | 0 | 0 | 0 | 0 | 1,347 | 2,072 | 60,623 | 58,551 |
| 5 | 29.63208 | -89.6627 | 0.74 | 2.7 | 8,239 | 0 | 0 | 10,390 | 0 | 3,620 | 229 | 0 | 22,478 | 119,592 | 97,113 |
| 6 | 29.77153 | -89.7306 | 0.96 | 2.1 | 698 | 0 | 0 | 498 | 302 | 0 | 0 | 559 | 1,498 | 27,039 | 25,541 |
| 7 | 29.63058 | -89.7109 | 0.74 | 2.7 | 806 | 9,222 | 0 | 0 | 2,834 | 0 | 203 | 0 | 13,066 | 98,885 | 85,819 |
| 8 | 29.83423 | -89.9522 | 1.04 | 2.0 | 3,053 | 0 | 236 | 0 | 0 | 0 | 0 | 251 | 3,289 | 37,249 | 33,960 |
| 9 | 29.58954 | -89.6134 | 0.94 | 2.1 | 883 | 0 | 1,568 | 667 | 1,236 | 0 | 0 | 7,037 | 4,354 | 159,820 | 155,466 |
| 10 | 29.63206 | -89.66 | 0.82 | 2.4 | 13,660 | 0 | 0 | 853 | 0 | 0 | 0 | 0 | 14,512 | 110,964 | 96,452 |
| 11 | 29.8147 | -89.775 | 0.47 | 4.3 | 53 | 0 | 0 | 0 | 0 | 382 | 0 | 3,917 | 435 | 15,837 | 15,403 |
| 12 | 29.60553 | -89.7456 | 0.94 | 2.1 | 10,178 | 5,871 | 0 | 0 | 1,924 | 0 | 0 | 2,125 | 17,972 | 87,362 | 69,390 |
| 13 | 29.78008 | -89.8429 | 0.91 | 2.2 | 0 | 1,048 | 0 | 0 | 1,042 | 0 | 0 | 3,885 | 2,090 | 24,090 | 22,000 |
| 14 | 29.61306 | -89.7849 | 0.66 | 3.0 | 2,895 | 0 | 0 | 6,683 | 0 | 0 | 3,038 | 1,505 | 12,615 | 84,550 | 71,935 |
| 15 | 29.74901 | -89.8243 | 0.64 | 3.1 | 0 | 0 | 0 | 2,076 | 0 | 0 | 0 | 7,544 | 2,076 | 64,457 | 62,381 |
| 16 | 29.62464 | -89.7844 | 0.70 | 2.9 | 3,946 | 556 | 0 | 0 | 7,701 | 4,338 | 0 | 2,624 | 16,541 | 108,378 | 91,837 |
| 17 | 29.76298 | -89.9327 | 0.24 | 8.3 | 0 | 0 | 0 | 0 | 0 | 0 | 4,954 | 0 | 4,954 | 23,110 | 18,156 |
| 18 | 29.62789 | -89.8147 | 0.74 | 2.7 | 5,812 | 0 | 0 | 6,553 | 0 | 0 | 0 | 879 | 12,365 | 65,770 | 53,405 |
| 19 | 29.62797 | -89.8241 | 0.91 | 2.2 | 0 | 3,842 | 393 | 0 | 0 | 0 | 0 | 680 | 4,235 | 40,633 | 36,398 |
| 20 | 29.63087 | -89.7152 | 0.73 | 2.7 | 16,306 | 0 | 0 | 632 | 0 | 0 | 0 | 0 | 16,937 | 123,683 | 106,746 |
| 21 | 29.71873 | -89.9183 | 0.82 | 2.4 | 0 | 1,014 | 0 | 3,556 | 1,810 | 945 | 0 | 0 | 7,325 | 64,005 | 56,680 |
| 22 | 29.66476 | -89.7503 | 0.96 | 2.1 | 5,610 | 98 | 0 | 203 | 200 | 0 | 0 | 400 | 6,111 | 88,800 | 82,689 |
| 23 | 29.70906 | -89.8053 | 0.73 | 2.7 | 2,250 | 0 | 0 | 6,847 | 0 | 0 | 2,176 | 0 | 11,274 | 65,996 | 54,722 |
| 24 | 29.7128 | -89.7258 | 1.00 | 2.0 | 2,942 | 0 | 0 | 4,621 | 0 | 0 | 0 | 0 | 7,563 | 60,874 | 53,311 |
| 25 | 29.67869 | -89.7028 | 0.87 | 2.3 | 4,054 | 0 | 0 | 4,748 | 0 | 379 | 0 | 139 | 9,181 | 81,297 | 72,116 |
| 26 | 29.6742 | -89.876 | 0.73 | 2.7 | 0 | 303 | 0 | 3,403 | 0 | 0 | 0 | 454 | 3,706 | 20,411 | 16,704 |
| 27 | 29.84385 | -89.813 | 0.82 | 2.4 | 286 | 16 | 0 | 0 | 627 | 0 | 0 | 1,138 | 930 | 20,118 | 19,188 |
